# The impact of margins and re‐resection in pediatric synovial sarcoma

**DOI:** 10.1002/cam4.70207

**Published:** 2024-09-15

**Authors:** Andres F. Espinoza, Priya B. Shetty, Jillian C. Jacobson, Hannah Todd, Kelley Harrell, Alfred F. Trappey, John Doski, Eumenia C. Castro, Nicole I. Montgomery, M. Fatih Okcu, Rajkumar Venkatramani, Dai H. Chung, Sanjeev A. Vasudevan

**Affiliations:** ^1^ Texas Children's Surgical Oncology Program and Liver Tumor Program, Division of Pediatric Surgery, Michael E. DeBakey Department of Surgery, Dan L. Duncan Cancer Center Baylor College of Medicine Houston Texas USA; ^2^ Department of Pediatric Hematology and Oncology Baylor College of Medicine and Texas Children's Hospital Houston Texas USA; ^3^ Section of Epidemiology and Population Sciences, Department of Medicine Baylor College of Medicine Houston Texas USA; ^4^ Division of Pediatric Surgery University of Texas Southwestern Medical Center and Children's Health Dallas Texas USA; ^5^ Division of Pediatric Surgery University of Texas San Antonio San Antonio Texas USA; ^6^ Department of Pathology and Immunology Baylor College of Medicine and Texas Children's Hospital Houston Texas USA; ^7^ Department of Orthopedics Baylor College of Medicine, Texas Children's Hospital and Cancer Center Houston Texas USA

**Keywords:** margins, pediatric, re‐resection, synovial sarcoma

## Abstract

**Introduction:**

Synovial sarcoma is one of the most common soft tissue sarcomas in children. Guidelines regarding the adequate extent of resection margins and the role of re‐resection are lacking. We sought to evaluate the adequate resection margin and the role of re‐resection in predicting outcomes in children with synovial sarcomas.

**Methods:**

A cohort of 36 patients less than 18 years of age at diagnosis who were treated for localized synovial sarcoma at three tertiary pediatric hospitals between January 2004 and December 2020 were included in this study. Patient and tumor demographics, treatment information, and margin status after surgical resection were collected from the medical record. Clinical, treatment, and surgical characteristics, as well as outcomes including hazard ratios (HRs), event‐free survival (EFS), and overall survival (OS) were compared by resection margins group and re‐resection status.

**Results:**

Patients in the R1 resection group were significantly more likely to relapse or die compared to patients in the R0 resection group. However, there was no significant difference in EFS (HR 0.52, *p* = 0.54) or OS (HR 1.56, *p* = 0.719) in R0 patients with less than 5 mm margins compared to R0 patients with more than 5 mm margins. Patients with R1 on initial or re‐resection had significantly worse OS than patients who had R0 resection on initial or re‐resection (HR = 10.12, *p* = 0.005).

**Conclusion:**

This study re‐affirms that R0 resection is an independent prognostic predictor of better OS/EFS in pediatric synovial sarcoma. Second, our study extends this finding to report negative margins on initial resection or re‐resection is associated with better OS/EFS than positive margins on initial resection or re‐resection. Lastly, we found that there is no difference in outcomes associated with re‐resection or <5 mm margins for R0 patients, indicating that re‐resection and <5 mm margins are acceptable if microscopic disease is removed.

## Graphical abstract

R0 resection is an independent prognostic predictor of better OS/EFS in pediatric synovial sarcoma. Negative margins on initial resection or re‐resection are associated with better OS/EFS than positive margins on initial resection or re‐resection. There is no difference in outcomes associated with re‐resection or  <5 mm margins for R0 patients.

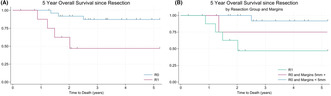

## 1 INTRODUCTION

Synovial sarcoma is the most common non‐rhabdomyosarcoma soft tissue sarcoma (STS).^1^, ^2^, ^3^ In the pediatric population, synovial sarcoma accounts for 10% of all STS.^1^, ^2^ The incidence of pediatric synovial sarcoma has been on the rise, aligning with climbing overall incidence of solid tumors among children.^2^, ^3^, ^4^ With this increase in incidence, the management of these aggressive tumors has evolved to require a multidisciplinary approach.^1^, ^2^, ^4^ Extensive surgical resection and aggressive medical approach is currently practiced as patients that relapse have been reported to have just 30% 5‐year survival rates.^1^, ^2^, ^3^, ^4^

Given the poor outcomes associated with relapsed disease, greater emphasis has been placed on studying the role of chemotherapy and radiation on the comprehensive management of these solid tumors. Recently, a landmark trial validated the role of perioperative chemotherapy and radiation, finding that combining both therapies result in event‐free survival rates of greater than 75% in those with intermediate and high risk tumors.^2^ Nonetheless, there remains a lack of agreement regarding the ideal resection margin.

Currently, physicians must decide between using the 1‐cm margin for STS as recommended by the National Comprehensive Cancer Network (NCCN) or the 0.5 cm margin as outlined by the Children's Oncology Group (COG) recommendations.^5^, ^6^, ^7^ The NCCN guidelines are primarily based on adult experiences, while the COG recommendations of margins have not been validated prospectively.^5^, ^6^, ^7^ Outcomes associated with re‐resection in patients with grossly negative margins but with positive microscopic margins (R1 resection) are presently unclear.^1^, ^3^ Adjuvant chemotherapy and radiation have been thoroughly validated to improve survival for patients with R1, but re‐resection and ideal surgical margins are left to single‐institution experiences.^1^, ^2^, ^3^

Without clear margin and re‐resection guidelines, technical surgical challenges arise when the tumor is located near neurovascular bundles or requires amputation. Intraoperatively, surgeons must decide whether to dissect out the tumor or to perform a more aggressive approach. The former risks the possibility of leaving microscopic disease behind, but the latter may require prosthesis or extensive reconstructions. We present a multi‐institutional analysis of localized pediatric synovial sarcoma patients to study the ideal margin required and the role of re‐resection to improve overall survival (OS).

## 2 MATERIALS AND METHODS

### 2.1 Patient population

We conducted a retrospective cohort study of children with localized synovial sarcoma who received care at three Texas‐based tertiary care, pediatric hospitals between January 2004 and December 2020. We reviewed the medical records of all patients under 18 years of age who had a pathology‐confirmed diagnosis of localized synovial sarcoma. All patients were treated with a combination of chemotherapy, radiation, and surgical resection according to their grade in each institution. Patient records were abstracted for data on demographics, disease, treatment, and outcomes. Recurrent disease was identified through cross‐sectional imaging and histological diagnosis on biopsy or recurrent resection. Patients were identified as either R1, meaning that they have grossly negative margins but positive microscopic margins, or R0, meaning that they have grossly negative margins and negative microscopic margins. The pathologist involved in all the cases performed a thorough evaluation of all the borders of the tumor. If there was concern designated by the surgeon, evaluation was performed intraoperatively with the pediatric surgeon indicating the area of concern and the pathologist evaluating through simply sectioning the tumor or frozen section procedure. The current study was approved and waiver of written consent was obtained by the Institutional Review Board (IRB) H‐50340 by Baylor College of Medicine, University of Texas at San Antonio, and University of Texas Southwestern.

### 2.2 Statistical analysis

Patient, tumor, and treatment characteristics were summarized in 36 patients with synovial sarcoma. The definition of an event was relapsed disease defined as recurrence of tumor after 4 weeks, progression of disease within 4 weeks, and death. Descriptive statistics, the chi‐squared test, and *t*‐tests were used to examine the relationships between these characteristics and the resection group (R0 vs. R1), margins group (<5 mm and ≥5 mm), and re‐resection group (yes vs. no). For the survival analysis, overall survival (OS) and event‐free survival (EFS) were calculated from the date of diagnosis to the date of the latest note available; deaths were scored as events and patients who were alive at the time of the most recent note were censored. The effect of different surgical approaches on OS was assessed with univariable Cox regression models, and hazard ratios with 95% confidence intervals (95% CIs) and *p*‐values were reported for these comparisons. Multivariable Cox regression analysis was used to evaluate differences in OS by resection group adjusted for radiation and chemotherapy, and these results were also reported with hazard ratios, 95% CI, and *p*‐values. Overall survival graphs were restricted to 5 years OS due to data sparsity. All analyses were performed using Stata SE18 (Statacorp, College Station, TX).

## 3 RESULTS

Thirty‐six patients with synovial sarcoma were included in this study. Twenty‐seven patients (75%) had a R0 resection (patients with grossly negative margins and negative microscopic margins), and 9 patients (25%) had a R1 resection (patients with grossly negative margins but positive microscopic margins). There was no significant difference between the groups by sex, race, ethnicity, or age at diagnosis (Table 1). Patients in the R1 group had significantly worse outcomes than patients in the R0 group. Specifically, R1 patients were more likely to have relapsed (88.9% vs. 29.6%, *p* = 0.002) or died (66.7% vs. 11.1%, *p* = 0.001) than patients in the R0 group resulting in a 5‐year EFS of 56.3% in R0 vs 0% in R1 (Figure S1A).

**TABLE 1:** Comparison of demographics, medical management, and outcomes by R0 and R1 resection cohort (*N* = 36).

	R0 (*n* = 27)	R1 (*n* = 9)	Chi‐squared test *p*‐value
Sex			
Female	11 (40.7%)	3 (33.3%)	0.693
Male	16 (59.3%)	6 (66.7%)	
Race			
Asian	1 (3.7%)	0 (0.0%)	0.235
Black or African American	6 (22.2%)	0 (0.0%)	
White	20 (74.1%)	9 (100.0%)	
Ethnicity			
Hispanic/Latinx	11 (40.7%)	4 (44.4%)	0.439
Not Hispanic/Latinx	16 (59.3%)	5 (55.6%)	
Relapse			
Yes	8 (29.6%)	8 (88.9%)	**0.002**
No	19 (70.4%)	1 (11.1%)	
Died			
Yes	3 (11.1%)	6 (66.7%)	**0.001**
No	24 (88.9%)	3 (33.3%)	
Tumor location			
Abdomen	2 (7.4%)	0 (0.0%)	**0.028**
Back	1 (3.7%)	0 (0.0%)	
Extremity	18 (66.7%)	2 (22.2%)	
Neck	1 (3.7%)	0 (0.0%)	
Thorax	5 (18.5%)	7 (77.8%)	
Tumor location			
Extremity	18 (66.7%)	2 (22.2%)	**0.020**
Not extremity	9 (33.3%)	7 (77.8%)	
Grade^a^			
2	16 (72.7%)	1 (16.7%)	**0.013**
3	6 (27.3%)	5 (83.3%)	
Radiation			
Neoadjuvant + adjuvant	2 (7.4%)	0 (0.0%)	**0.004**
Neoadjuvant	4 (14.8%)	1 (11.1%)	
Adjuvant	5 (18.5%)	8 (88.9%)	
None	15 (55.6%)	0 (0.0%)	
Unknown course	1 (3.7%)	0 (0.0%)	
Radiation—any versus none			
Yes	12 (44.4%)	9 (100.0%)	**0.003**
No	15 (55.6%)	0 (0.0%)	
Chemotherapy			
Neoadjuvant + adjuvant	3 (11.1%)	1 (11.1%)	**0.010**
Neoadjuvant	7 (25.9%)	3 (33.3%)	
Adjuvant	2 (7.4%)	5 (55.6%)	
None	14 (51.9%)	0 (0.0%)	
Unknown course	1 (3.7%)	0 (0.0%)	
Chemo—any versus none			
Yes	13 (48.2%)	9 (100.0%)	**0.006**
No	14 (51.9%)	0 (0.0%)	
Age at Dx			
Median; mean (range)	12; 11.5 (2, 16)	13; 12.1 (7, 16)	0.65 (*t*‐test)
Largest tumor dimension (mm)^b^			
Median; mean (range)	5.4; 6.7 (1.5, 23.6)	7.6; 8.7 (4.1, 18)	0.34 (*t*‐test)

*Note*: Bold values are statistically significant.

^a^ Eight patients were missing tumor grade information.

^b^ Three patients were missing tumor dimension information.

R1 patients' tumors were significantly less likely to be in the extremities compared to R0 patients' tumors (22.2% vs. 66.7%; *p* = 0.02). However, there was no significant difference in largest tumor dimension between the groups (*p* = 0.34). The R1 tumors were of higher grade than R0 tumors; 83.3% of R1 tumors were grade 3 compared to 27.3% of R0 tumors (*p* = 0.013). With regards to medical management, all of the R1 patients received radiation therapy and chemotherapy, whereas 44.4% of R0 patients received radiation therapy (*p* = 0.003) and 48.2% of R0 patients received chemotherapy (*p* = 0.006).

Among R0 patients, 78.3% (*n* = 18) had margins <5 mm compared to 21.7% of patients having ≥5 mm margins (*n* = 5). We repeated the analyses conducted in Table 1 for R0‐only patients, comparing by margins group (<5 mm vs. ≥5 mm, Table 2). We found that there was no significant difference in patient or clinical characteristics or outcomes for R0 patients with <5 mm margins compared to patients with ≥5 mm margins. Tumor grade showed the most notable difference between the two groups with ≥5 mm tumors being more likely to be grade 3 than <5 mm tumors, however this comparison was also not significant (75.0% vs. 26.7%; *p* = 0.075). These sub‐analyses were conducted on 23 patients from the cohort, as 14 patients were excluded due to being R1 (*n* = 9) or not having re‐resection information (*n* = 4).

**TABLE 2:** Comparison of demographics, medical management, and outcomes by margins cohort in R0 patients (*N* = 23).

	R0/margins <5 mm (*n* = 18)	R0/margins 5+ mm (*n* = 5)	Chi‐squared test *p*‐value
Sex			
Female	6 (33.3%)	2 (40.0%)	0.782
Male	12 (66.7%)	3 (60.0%)	
Race			
Asian	0 (0.0%)	1 (20.0%)	0.087
Black or African American	4 (22.2%)	2 (40.0%)	
White	14 (77.8%)	2 (40.0%)	
Ethnicity			
Hispanic/Latinx	7 (38.9%)	2 (40.0%)	0.964
Not Hispanic/Latinx	11 (61.1%)	3 (60.0%)	
Relapse			
Yes	5 (27.8%)	1 (20.0%)	0.726
No	13 (72.2%)	4 (80.0%)	
Died			
Yes	2 (11.1%)	1 (20.0%)	0.602
No	16 (88.9%)	4 (80.0%)	
Tumor location			
Abdomen	1 (5.6%)	0 (0.0%)	0.324
Back	0 (0.0%)	0 (0.0%)	
Extremity	12 (66.7%)	5 (100.0%)	
Neck	0 (0.0%)	0 (0.0%)	
Thorax	5 (27.8%)	0 (0.0%)	
Tumor location			
Extremity	12 (66.7%)	5 (100.0%)	0.133
Not Extremity	6 (33.3%)	0 (0.0%)	
Grade^a^			
2	11 (73.3%)	1 (25.0%)	0.075
3	4 (26.7%)	3 (75.0%)	
Radiation			
Neoadjuvant + Adjuvant	1 (5.6%)	0 (0.0%)	0.678
Neoadjuvant	4 (22.2%)	0 (0.0%)	
Adjuvant	3 (16.7%)	1 (20.0%)	
None	9 (50.0%)	4 (80.0%)	
Unknown course	1 (5.6%)	0 (0.0%)	
Radiation—Any versus None			
Yes	9 (50.0%)	1 (20.0%)	0.231
No	9 (50.0%)	4 (80.0%)	
Chemotherapy			
Neoadjuvant + Adjuvant	2 (11.1%)	0 (0.0%)	0.747
Neoadjuvant	5 (27.8%)	1 (20.0%)	
Adjuvant	1 (5.6%)	1 (20.0%)	
None	9 (50.0%)	3 (60.0%)	
Unknown course	1 (5.6%)	0 (0.0%)	
Chemo—Any versus None			
Yes	9 (50.0%)	2 (40.0%)	0.692
No	9 (50.0%)	3 (60.0%)	
Age at Dx			
Median; mean (range)	14; 11.7 (2, 16)	12; 11.2 (6, 16)	0.80 (*t*‐test)
Largest tumor dimension (mm)^b^			
Median; mean (range)	6.6; 5.9 (1.5, 13.1)	4.8; 7.6 (3.7, 17)	0.47 (*t*‐test)

^a^ 4 patients were missing tumor grade information.

^b^ 3 patients were missing tumor dimension information.

**FIGURE 1:**
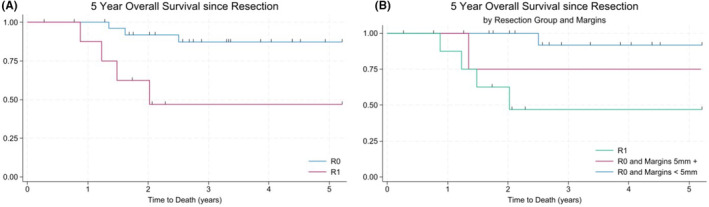
Overall survival comparing resection margin. (A) R0 resection patients had 8.66‐fold higher OS than those with R1 resection (*p* = 0.004). (B) Patients with R0 and margins >5 mm had a 1.56‐fold higher OS than those with R0 and margins <5 mm (*p* = 0.719). R0 *n* = 27, R1 (*n* = 9), R0 and margins 5 mm + (*n* = 5), R0 and margins <5 mm (*n* = 18).

In the survival analysis examining time since the resection, the 5‐year OS for R1 patients was significantly worse than for the R0 patients (log‐rank test *p* = 0.0007) (Figure 1A). Of the 6 deaths in the R1 group (*n* = 9), 3 occurred within the first 1.5 years following resection. In comparison, the R0 group (*n* = 27) had three deaths and only one of these occurred in the first 1.5 years following resection. Specifically, the patients who had a R1 resection had a HR of 8.66 (*p* = 0.004) of death compared to patients in the R0 group and HR of 2.86 for an event (*p* = 0.072) (Tables 3 and 4). However, this difference was muted when we controlled for radiation and chemotherapy when looking at OS (HR = 3.33, *p* = 0.111) and EFS (HR = 1.86, *p* = 0.324) in the multivariable Cox model, which is consistent with the findings of the STS landmark trial.^2^

**TABLE 3:** Univariable and multivariable overall survival analysis results by medical management and surgical approaches.

Comparison	Hazard ratio	Standard error	95% CI	*p*‐value
R1 versus R0	8.66	6.54	(1.97, 38.04)	0.004
Tumor location: extremity versus not	0.20	0.17	(0.04, 1.04)	0.06
Radiation: any versus none	^a^			
Chemotherapy: any versus none	^b^			
R0 ≥5 mm margins versus R0 <5 mm margins	1.56	1.94	(0.14, 17.75)	0.719
Positive margins (R1 or on rre‐resection) versus Negative margins (R0 or on re‐resection)	10.12	8.34	(2.01, 50.85)	0.005
R1 versus R0, controlling for radiation (any vs. none) and chemotherapy (any vs. none)	3.33	2.51	(0.76, 14.6)	0.111

^a^ All patients who died received radiation.

^b^ All patients who died received chemotherapy.

**TABLE 4:** Univariable and multivariable EFS analysis results by medical management and surgical approaches.

Comparison	Hazard ratio	Standard error	95% CI	*p*‐value
R1 versus R0	2.86	1.67	(0.91, 8.99)	0.072
Tumor location: extremity versus not	0.30	0.17	(0.10, 0.90)	0.031
Radiation: any versus none	3.21	1.97	(0.96, 10.7)	0.06
Chemotherapy: any versus none	2.67	1.60	(0.83, 8.62)	0.10
R0 ≥5 mm margins versus R0 <5 mm margins	0.52	0.56	(0.06. 4.30)	0.54
Positive margins (R1 or on re‐resection) versus Negative margins (R0 or on re‐resection)	3.19	1.82	(1.05, 9.73)	0.041
R1 versus R0, controlling for radiation (any vs. none) and chemotherapy (any vs. none)	1.86	1.18	(0.54, 6.43)	0.324

**FIGURE 2:**
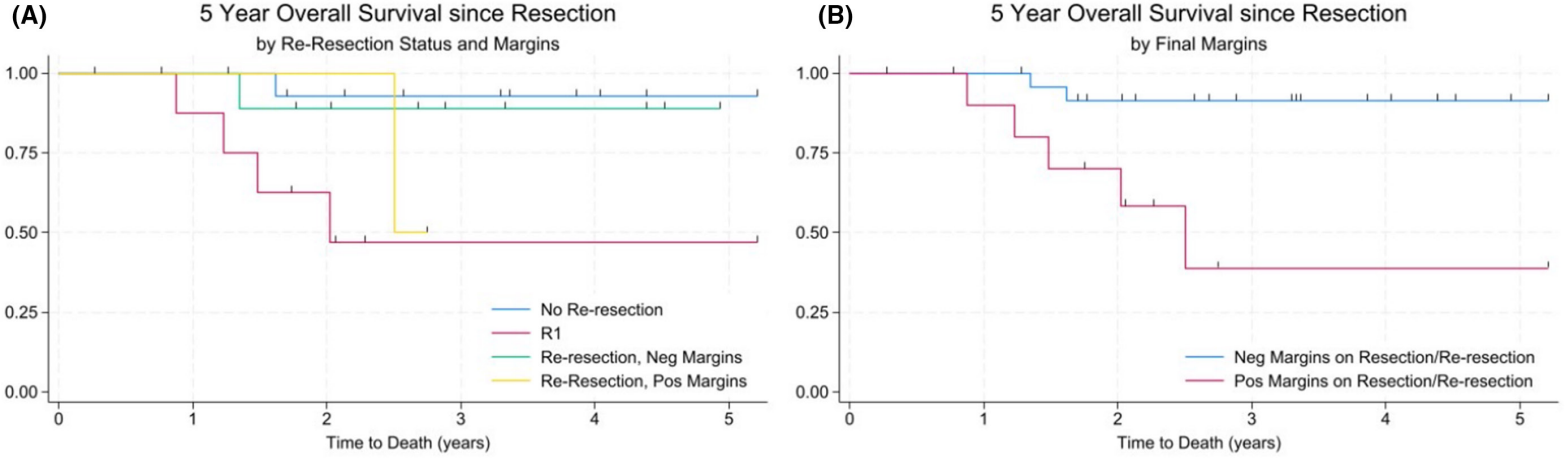
Overall survival in patients that underwent re‐resection. (A) Patients who were R0 on initial surgery had a 1.6‐fold higher OS than those with re‐resection that achieved negative margins (*p* = 0.72). (B) OS was 10.12‐fold higher in those with negative margins compared to those with positive margins after re‐resection was attempted (*p* = 0.005). No re‐resection (*n* = 21), R1 *n* = 4, re‐resection, negative Margins (*n* = 6), re‐resection positive margins (*n* = 5), negative margins on resection/re‐resection (*n* = 27), positive margins on resection/re‐resection (*n* = 9).

Focusing on the R0 group, we found that there was no significant difference in OS (HR = 1.56, *p* = 0.719) or EFS (0.52, *p* = 0.54) in the R0 group between those that had <5 mm margins and those with ≥5 mm margins (Figure 1B, Tables 3 and 4, Figure S1B). OS and EFS did not differ significantly between those that had R0 resection on initial resection compared to those that had R0 on re‐resection (Figure 2A, Figure S2A). Patients who had positive margins on initial or re‐resection had 10.12‐fold worse OS (*p* = 0.005) and 3.19‐fold worse EFS (*p* = 0.041) than patients who had negative margins on initial or re‐resection (Figure 2B, Tables 3 and 4, Figure S2B), suggesting that considering re‐resection to achieve negative margins may be worthwhile for improved OS and EFS.

## 4 DISCUSSION

Synovial sarcoma is one of the most common soft tissue sarcomas in children.^1^ Previous studies on pediatric synovial sarcoma have demonstrated that OS is improved when R0 resection is achieved.^1^, ^2^, ^3^, ^4^ Despite this, clinicians currently decide on margin size and re‐resection based on single institutional experience due to the lack of clear guidelines. This study contributes to the literature by providing supporting evidence that R0 resection improves long‐term survival, whether it is achieved initially or on re‐resection. In addition, we show that re‐resection and <5 mm margins improve OS if R0 resection is achieved.

Our patient cohort with R0 resection had significantly better OS than those with R1 resection. While not evaluated in the present study, other multi‐institutional studies have found that the use of perioperative chemotherapy and radiation is an independent factor that improves survival and reduces recurrence.^2^, ^3^, ^4^ In those patients where R1 resection is achieved, chemotherapy and radiation were found to significantly reduce the incidence of recurrence and was associated with improved OS.^2^, ^3^, ^4^, ^5^, ^6^ Among patients receiving radiation, chemotherapy, or both, patients in the R1 group had 3.3 times worse OS (*p* = 0.111) and 1.86 worse EFS (*p* = 0.324) than patients in the R0 group. Although this difference was marginally not statistically significant, it was a strong effect size for a small sample size and suggests that further study is needed. Regardless of this finding, we feel that this study confirms with the literature that extent of margins is not a key factor in affecting survival, simply achieving an R0 resection maybe adequate in obtaining the best survival, and R1 resection patients may be salvaged to some degree with adjuvant chemotherapy and radiation therapy. Of note over half of the patients that achieved an R0 resection did not receive adjuvant therapies and still attained good EFS and OS.

While R0 resection has been established to improve survival, the need for a specific margin is under‐studied. There are inconsistent recommendations concerning margins not only across individual continents but also across different specialties and by professional groups such as COG and NCCN.^5^, ^6^, ^7^, ^8^, ^9^, ^10^, ^11^ In addition, apart from the quantitative evaluation of margins that have proved to be crucial, there is limited data that guides how aggressive to be when tumors approach or go through membrane‐like structures such as periosteum or fascia, Our experience demonstrates that regardless of the location of the tumors R0 patients that had <5 mm margins had similar, if not improved, outcomes compared to those with ≥5 mm margins. This finding further illustrates the importance of elimination of microscopic disease while recognizing that removing additional benign tissue does not improve patient outcomes.

As recent studies have validated that frozen sections are both specific and sensitive in soft tissue sarcomas, this may prove to be a useful tool for surgeons intraoperatively.^12^, ^13^ In addition, evidence shows pediatric frozen sections are underutilized, which emphasizes the need for further studies on this vulnerable population.^12^ To also aid in obtaining a R0 resection, intraoperative imaging may prove to be useful and is currently being investigated. Intraoperative ultrasound and imaged guided fluorescence have been shown to reduce the risk of positive margin status in soft tissue sarcomas.^14^, ^15^ Given this, further prospective randomized clinical trials are needed to validate this finding in pediatric synovial sarcoma.

As the literature on re‐resection in synovial sarcoma is primarily focused on adult patients, this study makes notable contributions to the literature in pediatric patients. The majority of studies evaluating pediatric re‐resection is in the setting of unplanned resection of synovial sarcoma, and they are biased towards surgeons that could not focus on obtaining an R0 margin.^16^, ^17^ In these patients, re‐resection attempts had high rates of positive margins, resulting in high rates of relapse and death. Despite this, metanalysis studies in adults have demonstrated that a multidisciplinary discussion and approach to re‐resection can result in 5‐year OS of >95%.^16^, ^17^

The only study, to our knowledge, that focused on pediatric re‐resection of several STS was able to result in the complete remission of approximately 85% of patients.^18^ We report here the patients that underwent re‐resection, and obtained R0 on re‐resection, had similar OS as those with initial R0 resection. Considering these findings, we believe that re‐resection should be considered in all patients who have R1 resection, given the improved survival that we reported. As re‐resection may involve performing a more radical approach with the risk of still not obtaining a R0 margin, pre‐operative counseling is imperative. As we found that patients that had re‐resection and still had R1 margins in re‐resection did not have improved survival, the risks should be thoroughly discussed with the patients and their families.

This study has key limitations that should be noted. The study is a retrospective analysis of non‐randomized patients, despite it presenting one of the largest cohorts of surgically focused pediatric synovial sarcoma in the United States. As a rare disease, the number of patients available for the study is limited and thus so is our ability to generalize recommendations from our results. Our extensive study period accounts for 16 years of experience across three large pediatric cancer centers, during which some of the treatment regimens and approaches may have evolved. One limitation to note is that all surgeries including primary resection and re‐resection were performed by pediatric surgeons that were managed with a planned resection. We did not have any case where patients were resected in an unplanned excision, which may be an interesting scenario to evaluate in future work. In addition, several of these patients were initially diagnosed at an outside hospital in which case, despite extensive documentation, may be subject to some incomplete records. Lastly, while our study demonstrates the significant difference that negative margins make on outcomes, it is under‐powered to determine whether adjuvant therapy can replace re‐resection in cases of R1 margins. However, our findings in this study indicates that after multi‐center validation there are opportunities for improved treatment and outcomes for pediatric synovial sarcoma patients.

We present one of the largest multi‐institution surgical studies that describes outcomes for pediatric patients who have localized synovial sarcoma. These tumors require a multidisciplinary approach with the goal of obtaining an R0 resection, even if the margins are <5 mm, to optimize OS. We have shown that eliminating microscopic disease is required and re‐resection should be considered in all patients to help improve survival.

## AUTHOR CONTRIBUTIONS

**Andres F. Espinoza:** Conceptualization (lead); data curation (lead); formal analysis (lead); funding acquisition (equal); investigation (equal); methodology (equal); resources (equal); writing – original draft (lead). **Priya B. Shetty:** Data curation (equal); formal analysis (equal); methodology (equal); resources (equal); supervision (equal); validation (equal); visualization (equal); writing – original draft (equal); writing – review and editing (equal). **Jillian C. Jacobson:** Data curation (equal). **Hannah Todd:** Data curation (equal). **Kelley Harrell:** Data curation (equal). **Alfred F. Trappey:** Data curation (equal). **John Doski:** Data curation (equal). **Eumenia C. Castro:** Data curation (equal). **Nicole I. Montgomery:** Conceptualization (equal); data curation (equal). **M. Fatih Okcu:** Conceptualization (equal); investigation (equal). **Rajkumar Venkatramani:** Conceptualization (equal); investigation (equal). **Dai H. Chung:** Conceptualization (equal); investigation (equal). **Sanjeev A. Vasudevan:** Conceptualization (lead); data curation (lead); formal analysis (lead); funding acquisition (lead).

## CONFLICT OF INTEREST STATEMENT

The authors declare no conflicts of interest.

## ETHICS STATEMENT

Ethical approval and waiver of written consent was obtained through Institutional Review Board (IRB) H‐50340 by Baylor College of Medicine, University of Texas at San Antonio, and University of Texas Southwestern.

## Supporting information


Data S1.

## Data Availability

Data sharing is not applicable to this article as no new data were created or analyzed in this study.
