# Supplementary material for: The impact of margins and re‐resection in pediatric synovial sarcoma
Source: Cancer Med. 2024 Sep 15;13(17):e70207. doi: 10.1002/cam4.70207 (PMC11403124; doi:10.1002/cam4.70207)
Supplement: Supplementary file 1 — Data S1. [file CAM4-13-e70207-s001.pdf]

Figure 1

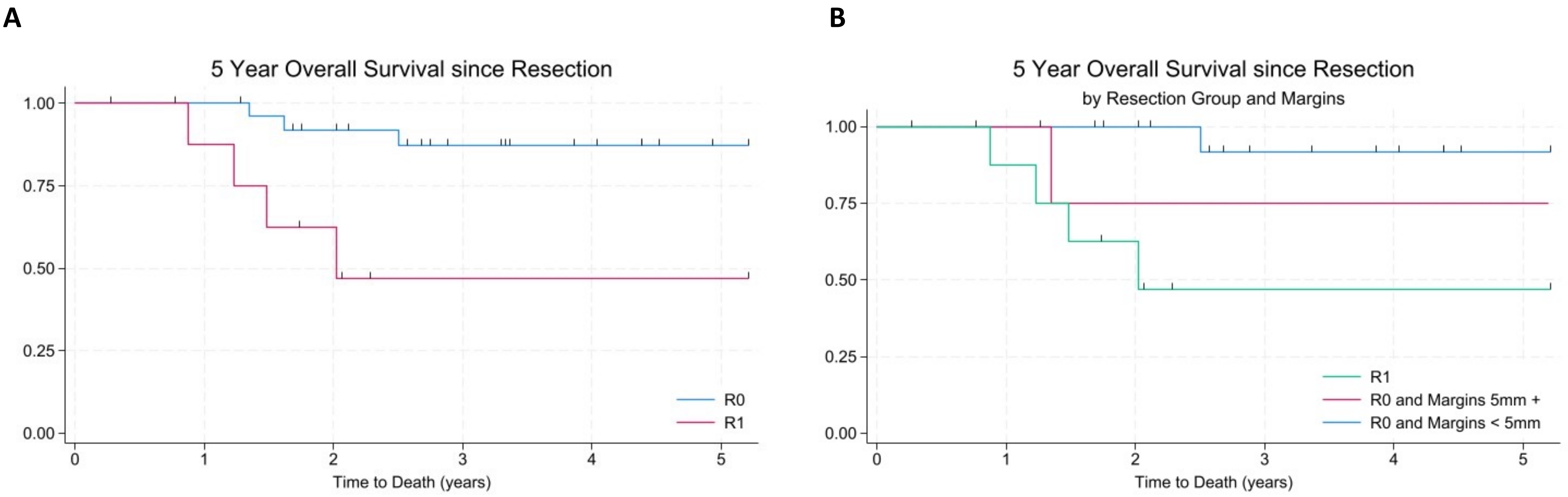

Figure 1. Overall Survival Comparing Resection Margin. (A) R0 resection patients had 8.66-fold higher OS than those with R1 resection (p=0.004). (B) Patients with R0 and margins $\geq$ 5mm had a 1.56-fold higher OS than those with R0 and margins < 5mm (p=0.719).

R0 n=27, R1 (n=9), R0 and margins 5 mm + (n=5), R0 and margins < 5mm (n=18)

Figure 2

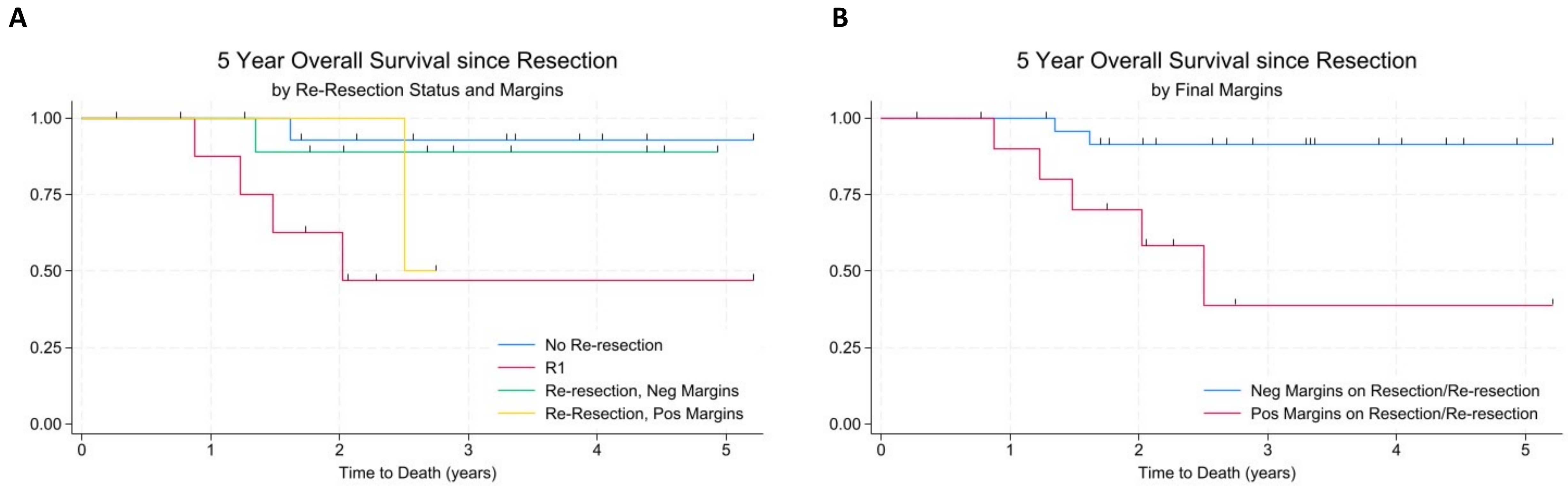

Figure 2. Overall Survival In Patients that Underwent Re-resection. (A) Patients who were R0 on initial surgery had a 1.6-fold higher OS than those with re-resection that achieved negative margins (p=0.72). (B) OS was 10.12-fold higher in those with negative margins compared to those with positive margins after re-resection was attempted (p=0.005).

No Re-resection (n=21), R1 n=4, Re-resection, Neg margins (n=6), Re-Resection Pos Margins (n=5), Neg Margins on Resection/Re-resection (n=27), PosMargins on Resection/Re-resection (n=9)

Supplementary Figure 1

A

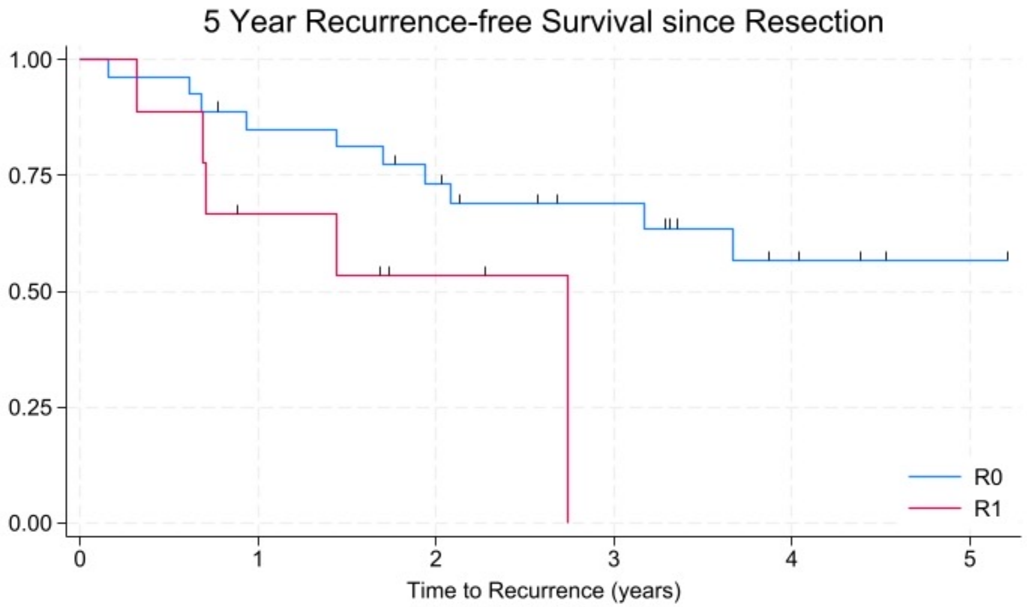

B

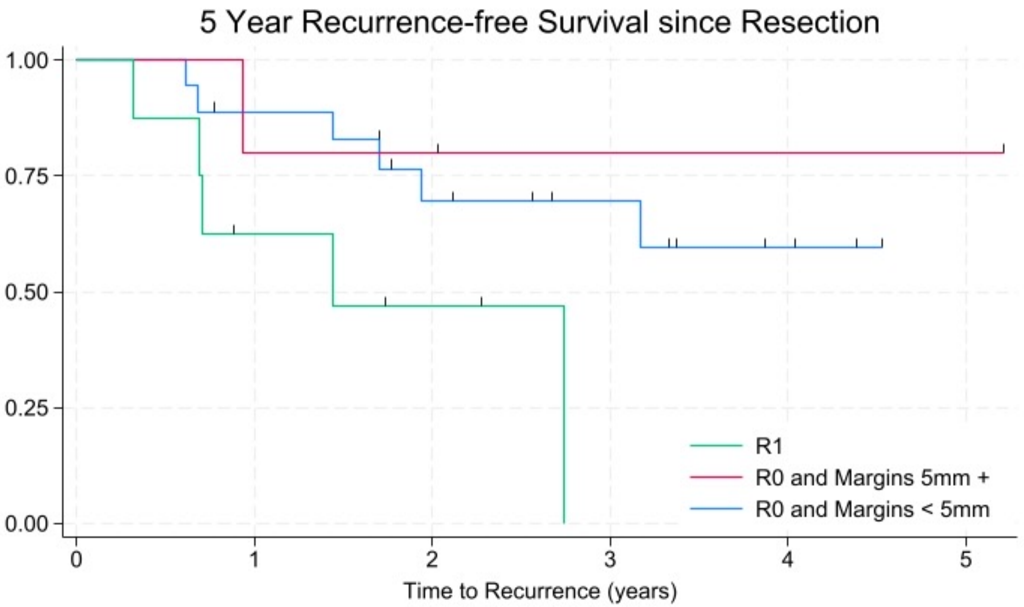

Supplementary 1. Event-free Survival Comparing Resection Margin. (A) R0 resection patients had 2.86 higher EFS than those with R1 resection (p=0.072). (B) Patients with R0 and margins $\geq$ 5mm had a 1.92-fold higher EFS than those with R0 and margins <5mm (p=0.54).

R0 n=27, R1 (n=9), R0 and margins 5 mm + (n=5), R0 and margins < 5mm (n=18)

Supplementary Figure 2

A

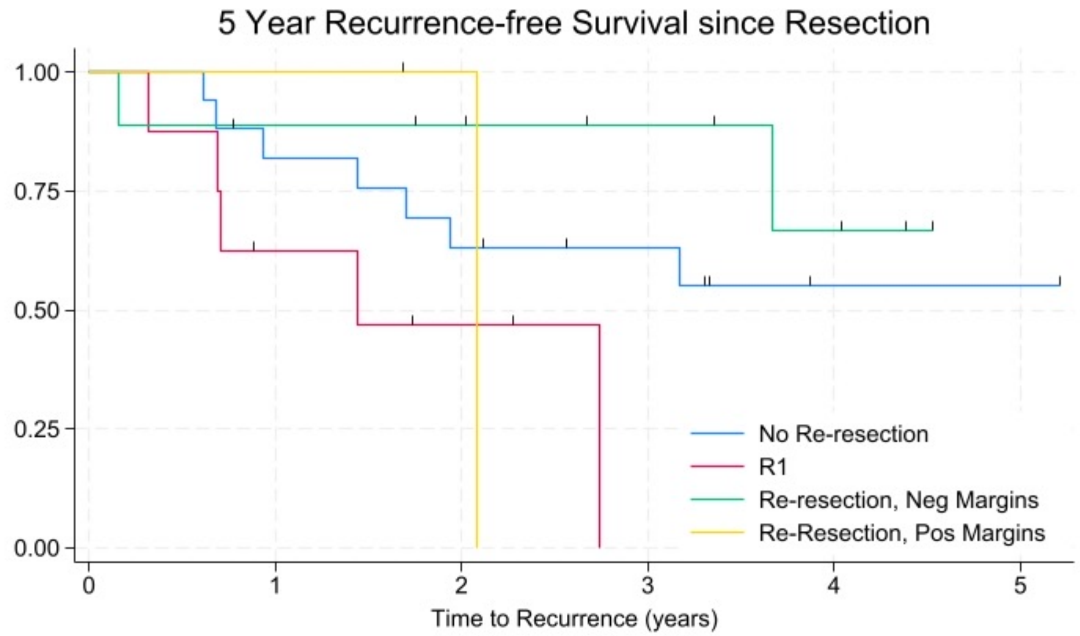

B

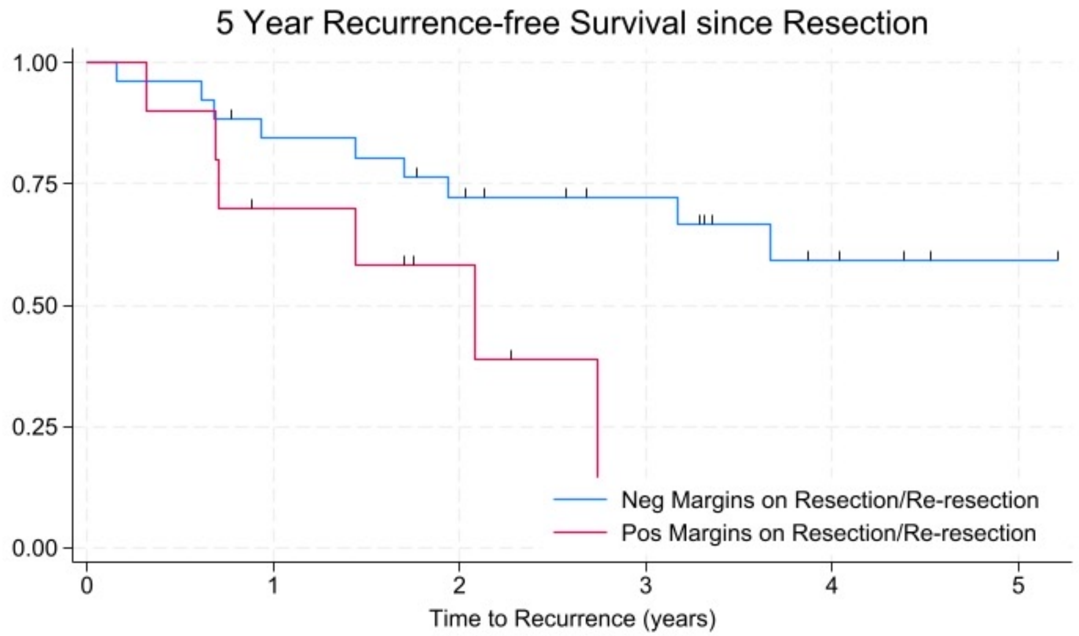

Supplementary 2. Event-free Survival in Patients that Underwent Re-resection. (A)) Patients who were R0 on initial surgery had a 2.09-fold higher EFS as those with re-resection that achieved negative margins (p=0.36). (B) OS was 3.19-fold higher in those with negative margins compared to those with positive margins after re-resection was attempted (p=0.041).

No Re-resection (n=21), R1 n=4, Re-resection, Neg margins (n=6), Re-Resection Pos Margins (n=5), Neg Margins on Resection/Re-resection (n=27), PosMargins on Resection/Re-resection (n=9)
